# International variations in chronic kidney disease patients’ pain experience and its management

**DOI:** 10.1093/ckj/sfaf346

**Published:** 2025-12-11

**Authors:** Rupesh Raina, Nikhil Nair, Rohan Kumar, Kush Doshi, Natalia Alencar-de Pinho, Charlotte Tu, Brian Bieber, Sophie Liabeuf, Christian Combe, Helmut Reichel, Christos Argyropoulos, Murilo Guedes, Roberto Pecoits-Filho

**Affiliations:** Department of Nephrology, Akron Children’s Hospital, Akron, OH USA; Akron Nephrology Associates/Cleveland Clinic Akron General Medical Center, Akron, OH USA; Case Western Reserve University School of Medicine, Cleveland, OH USA; University School, Hunting Valley, OH USA; Akron Nephrology Associates/Cleveland Clinic Akron General Medical Center, Akron, OH USA; Center for Research in Epidemiology and Population Health, Paris-Saclay University, Paris-Sud University, Versailles Saint Quentin University, INSERM, Villejuif, France; Arbor Research Collaborative for Health, Ann Arbor, MI USA; Arbor Research Collaborative for Health, Ann Arbor, MI USA; Pharmacoepidemiology Unit, Department of Clinical Pharmacology, Amiens-Picardie University Medical Center, Amiens, France; MP3CV Laboratory, Jules Verne University of Picardie, Amiens, France; Service de Néphrologie Transplantation Dialyse Aphérèse, CHU de Bordeaux, Univ. Bordeaux, Bordeaux, France; Nephrological Center, Villingen Schwenningen, Germany; Division of Nephrology, University of New Mexico School of Medicine, University of New Mexico, Albuquerque, NM USA; Department of Medicine, Stanford University, Stanford, CA USA; Arbor Research Collaborative for Health, Ann Arbor, MI USA

**Keywords:** analgesic, CKD, opioids, pain, quality of life

## Abstract

**Background:**

Chronic pain significantly impacts health-related quality of life (HRQOL) in patients with non-dialysis chronic kidney disease (ND-CKD), yet the management of pain in this population is challenging. We hypothesized that analgesic prescription practices vary internationally, influencing the pain experience and HRQOL of patients with stage 3–5 ND-CKD.

**Methods:**

This descriptive, observational, multinational cohort study utilized data from the Chronic Kidney Disease Outcomes and Practice Patterns Study (CKDopps), enrolling adult patients from nephrology practices in Brazil, France and the USA between 2013 and 2020. Analgesic prescriptions within 6 months before HRQOL assessment were categorized as non-steroidal anti-inflammatory drugs (NSAIDs), opioids or other analgesics. HRQOL was measured using the Kidney Disease Quality of Life Short Form, assessing multiple subdomains.

**Results:**

Among 3945 patients, analgesics were most frequently prescribed in the USA across all CKD stages, with opioids prescribed nearly twice as often compared with Brazil and France. NSAIDs are frequently prescribed in Brazil, including in advanced CKD stages, contrasting sharply with practices in France and the USA. Higher reported pain intensity consistently correlated with poorer outcomes across all HRQOL subdomains.

**Conclusions:**

This study identifies considerable international variability in pain reporting and analgesic prescription patterns in patients with stage 3–5 ND-CKD. Randomized controlled trials evaluating the efficacy and safety of analgesics are warranted to improve key patient-reported outcomes such as pain in patients with ND-CKD.

KEY LEARNING POINTS **What was known:**

- Pain is common among non-dialysis chronic kidney disease (ND-CKD) patients and its treatment is considered a priority for improving patient-centred outcomes in CKD.
- Significant variations exist in analgesic prescribing practices internationally, with limited understanding of how these variations relate to patient-reported pain levels.
- Previous observational studies lacked standardized patient-reported outcomes on pain, complicating comparisons across different healthcare settings and countries.

**This study adds:**

- Clear international differences in analgesic prescribing were identified; opioid use is notably higher in the USA compared with Brazil and France, even among patients reporting mild pain.
- Non-steroidal anti-inflammatory drugs (NSAIDs) are frequently prescribed in Brazil, including in advanced CKD stages, despite their known risks, contrasting sharply with practices in France and the USA.
- Increased pain severity consistently correlated with poorer health-related quality of life across physical and emotional domains, highlighting the urgent need for improved pain management strategies.

**Potential impact:**

- These findings underscore the need for international guidelines to standardize pain management practices in CKD patients, potentially reducing practice variations and associated adverse outcomes.
- Healthcare providers should be vigilant about regional differences in analgesic use and their implications for patient safety, particularly regarding NSAID use in advanced CKD.
- This study supports integrating systematic patient-reported outcomes in clinical care, enhancing individualized management of chronic pain in nephrology practice.

## INTRODUCTION

Pain is a common and debilitating symptom that is often observed in patients with chronic kidney disease (CKD) [1]. The occurrence of pain increases with CKD progression and moderateto severe pain is reported in 37–50% of patients on dialysis treatment [2–4]. Uncontrolled pain adversely affects health-related quality of life (HRQOL), including depression, anxiety, fatigue and healthcare utilization [5–8]. Pain management is considered a priority for patients with CKD [9].

Adequate control of pain is a clinical challenge in CKD patients. As the estimated glomerular filtration rate (eGFR) declines, the risks of adverse reactions to several analgesics increase, potentially leading to inappropriate treatment of pain in the advanced CKD population [10]. Medications that are commonly prescribed in the general population, such as non-steroidal anti-inflammatory drugs (NSAIDs), should be avoided in CKD due to the high incidence of adverse events, particularly the increased risk of CKD progression and acute kidney injury events. Safety concerns may vary from an individual prescriber perspective, leading to practice variation across regions in the world, especially in the absence of international guidelines on pain management in CKD.

Among non-dialysis CKD (ND-CKD) patients, the use of analgesics is common and seems to vary considerably across regions of the world. A systematic review and meta-analysis estimated a prevalence of ≈51% of analgesic use in ND-CKD, with considerable variation by region, particularly for NSAID use [11]. In this meta-analysis, opioid use tended to be more often reported (24%) compared with NSAIDs (17%) [11]. However, interpreting these pooled results is difficult due to the large heterogeneity in the estimates, which can stem from both clinical and methodological variations across observational studies. Lack of standardized data collection on medications can lead to important variations in prevalence estimates for analgesic use. Another frequent limitation of prior observational studies evaluating practice patterns for analgesic prescriptions in ND-CKD is the lack of data on patient-reported outcomes, particularly pain. The perception and reporting of pain are complex and can be affected by sociocultural factors and social determinants of health [12]. Moreover, the lack of comparative data on the safety and effectiveness of analgesics in the CKD population further limits prior studies. The extent to which the prescription of analgesics varies among ND-CKD patients across countries is as yet undetermined in the literature. The CKD Practice Patterns and Outcomes Study (CKDopps) is a prospective study designed to capture practice patterns and clinical and patient-reported outcomes in different countries following a standardized protocol for data collection.

In this international observational study among ND-CKD patients under nephrology care, we seek to describe country differences in patient-reported pain levels and the prescription of analgesics. We report the use of analgesics stratified by self-reported pain and associations with HRQOL in a multinational representative study with standardized data collection across countries.

## MATERIALS AND METHODS

### Data source

The CKDopps is an ongoing prospective cohort study of stage 3–5 ND-CKD adult patients treated in nephrologist-led CKD clinics in Brazil, France, Germany, Japan and the USA. In each country, a national list of nephrologist-run clinics was assembled to serve as the sampling frame. Random selection of clinics was stratified by geographic region and key clinic characteristics (e.g. clinic size and public versus private) to be as nationally representative as possible. Within each stratum, randomly selected clinics were sequentially approached for participation. Included patients were at least 18 years of age (without an upper age limit), receiving care for CKD at the clinic and had an eGFR <60 ml/min/1.73 m^2^ at the time of screening. The CKDopps study design, details and objectives have previously been published [13]. CKDopps was approved by national and/or local ethics committees and patient consent was obtained as required by local ethics regulations.

### Patient sample

This is a cross-sectional, descriptive study using data from the CKDopps cohort. Our analysis includes countries for which data were available regarding practice patterns for pain management thus we included patients from Brazil, France and the USA enrolled in the CKDopps study between 2013 and 2020. We excluded patients without information on medical prescriptions and the pain question from the Kidney Disease Quality of Life Short Form (KDQOL-SF).

### Study variables

#### Self-reported pain

We assessed self-reported pain by the pain question from the KDQOL-SF questionnaire. The 12-item KDQOL-SF is a validated instrument combining the general HRQOL and kidney-specific questions for patients with significant kidney comorbidities [14]. Patients answered the following pain question in the KDQOL-SF: ‘During the past 4 weeks, how much did pain interfere with your normal work (including both outside the home and housework)?’. The five response options were 1, not at all; 2, a little bit; 3, moderately; 4, quite a bit; and 5, extremely.

#### Analgesic prescriptions

We categorized the analgesic drugs into NSAIDs, opioids and others. Aspirin, acetaminophen and metamizole were not considered NSAIDs in any country. Information on analgesic prescriptions was assessed within 6 months of the HRQOL assessments conducted in the study. Patients without HRQOL assessments were excluded from the study.

#### KDQOL-SF

We analysed the following subdomains of the KDQOL-SF: emotional role, emotional well-being, energy, general health, physical functioning, physical role and social functioning. We did not use the mental component score (MCS) and physical component score (PCS) because our pain exposure variable is one of the components used to calculate these scores.

### Statistical analysis

Standard descriptive statistics [means and standard deviations (SDs) or medians and interquartile ranges (IQRs) for continuous variables and frequencies for categorical variables] were used to report patient characteristics by country and by CKD stages 3a, 3b and 4–5 (i.e. eGFR 45–59, 30–44, 15–29 and <15 ml/min/1.73 m^2^, respectively). All statistical analyses were conducted with SAS software (version 9.4; SAS Institute, Cary, NC USA). Standard descriptive statistics are used to explore patient characteristics (e.g. demographics, comorbidities, laboratory values, prescriptions) categorized by overall pain intensity and analgesic description. We also reported analgesic prescriptions by country, CKD stage and pain intensity.

## RESULTS

**Figure 1:**
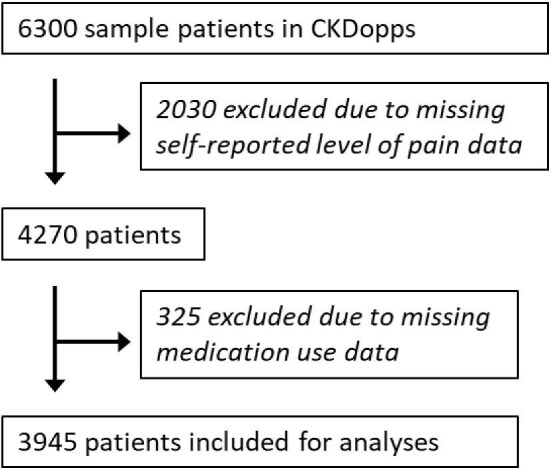
Assembly of the study cohort.

**Figure 2:**
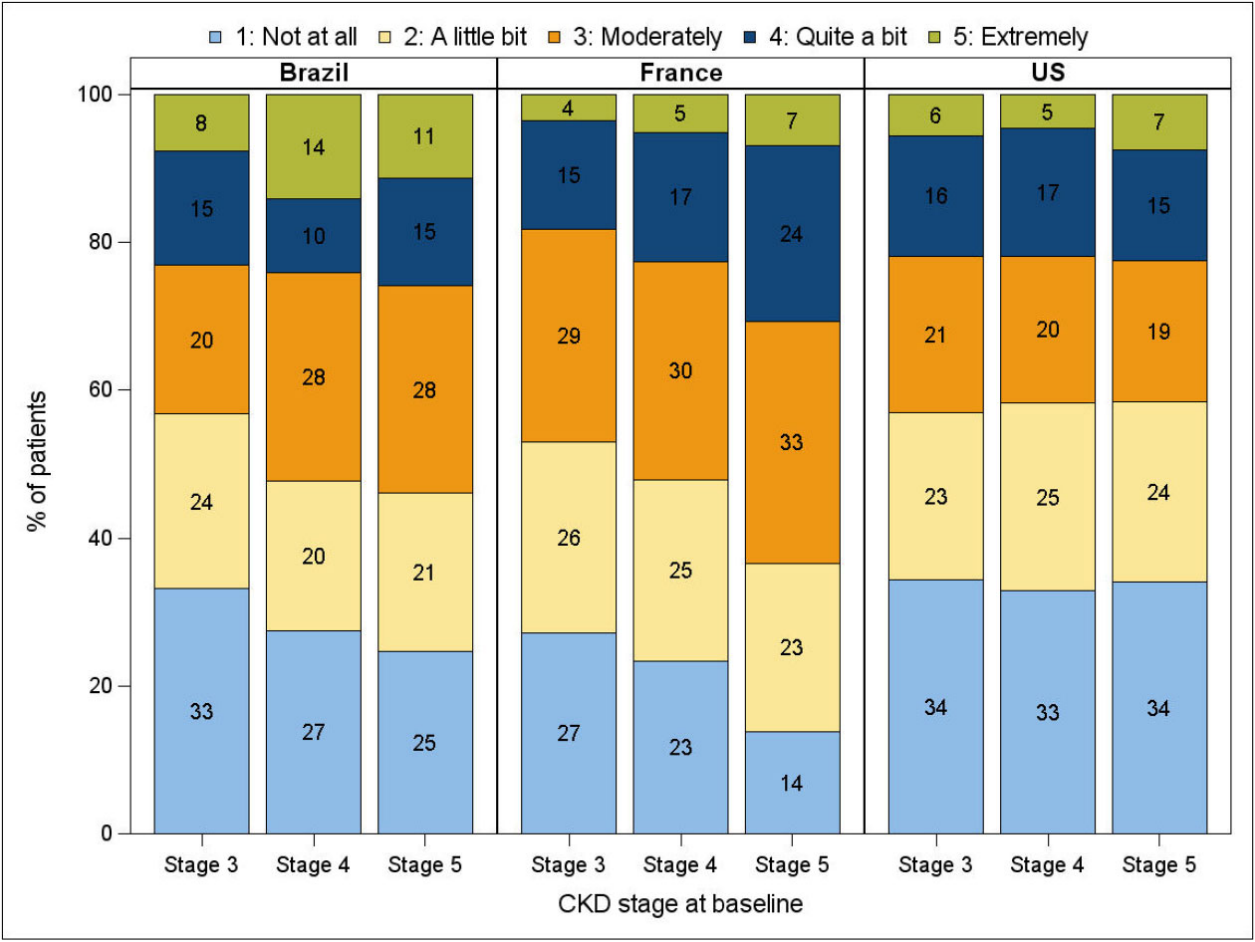
Pain levels by percentage across CKD stages.

A total of 3945 patients (Fig. 1) were included in this study, as depicted in Table 1. Overall, patients had a mean age of 68 years, were mostly male (59%) and had a mean eGFR of 30.2 ml/min/1.73 m^2^ at study entry. The most reported causes of CKD were hypertension (26%), followed by diabetes (25%) and glomerulonephritis/vasculitis (16%). The most common comorbidities were hypertension (88%), diabetes (71%) and congestive heart failure (26%). Mean haemoglobin and C-reactive protein (CRP) at study entry were 12.7 g/dl and 3.8 mg/dl, respectively. The distribution of pain by CKD stage across countries is depicted in Fig. 2. Patient characteristics varied by baseline pain levels. Patients who reported higher pain levels tended to be older, have a higher body mass index, lower eGFR and were more often female (Table 1). The number of comorbidities tended to be higher in the group reporting more severe pain, especially diabetes, peripheral artery disease and psychiatric or neurological disorders. Finally, CRP levels were slightly higher in the subgroup reporting extreme levels of pain compared with those reporting no pain at all.

**Table 1:** Patient characteristics at baseline by pain levels.

Characteristics	Not at all	A little bit	Moderately	Quite a bit	Extremely
Patients, *n*	1097	976	1031	624	217
Demographics					
Age (years), mean (SD)	64.9 (14.0)	67.3 (12.5)	69.2 (11.9)	69.0 (11.5)	70.1 (12.1)
Female, %	35	37	42	47	58
BMI (kg/m^2^), median (IQR)	26.8 (24.2–30.4)	28.0 [24.7,31.6]	28.6 [25.2,32.8]	30.7 [26.3,35.6]	30.9 [26.0,36.7]
Characteristics of CKD					
eGFR (ml/min/1.73 m^2^), mean (SD)	30.8 (12.1)	30.4 (11.6)	30.2 (11.4)	29.9 (11.3)	27.6 (10.5)
CKD stage %					
Stage 3	50	48	47	45	37
Stage 4	44	46	45	47	51
Stage 5	7	7	8	8	12
Reported cause of CKD, %					
Diabetes	22	21	26	30	43
Hypertension	24	25	27	29	23
Glomerulonephritis/vasculitis	19	16	15	13	10
Tubulointerstitial disease	13	13	12	10	12
ADPKD	6	7	5	3	5
Other	11	12	11	8	3
Unknown	5	7	5	7	2
Comorbidities, %					
Congestive heart failure	19	25	28	35	36
Cerebrovascular disease	9	8	13	15	16
Other cardiovascular disease	19	22	29	29	33
Peripheral vascular disease	13	16	24	24	29
Hypertension	86	88	90	90	93
Diabetes	62	68	76	79	71
Cancer (non-skin)	19	20	23	21	14
Gastrointestinal bleeding	2	3	4	4	2
HIV infection	2	0	1	1	1
Lung disease	7	10	12	16	16
Neurologic disease	3	3	4	4	9
Any psychiatric disorder	6	10	12	16	22
Recurrent cellulitis/gangrene	3	3	5	6	6
Laboratory					
Haemoglobin (g/dl) mean (SD)	12.9 (1.8)	12.8 (1.8)	12.7 (1.7)	12.3 (1.7)	12.1 (1.7)
CRP (mg/dl), median (IQR)	3.0 (1.0–5.3)	3.1 (1.8–7.3)	4.0 (1.6–8.3)	5.0 (2.3–13.3)	5.2 (2.8–13.2)
AST (U/l), median (IQR)	20.0 (16.0–25.0)	20.5 (17.0–26.0)	20.0 (17.0–26.0)	21.0 (16.0–26.0)	20.0 (15.0–29.0)
ALT (U/l), median (IQR)	18.0 (13.0–26.0)	19.0 (15.0–25.0)	18.0 (14.0–26.0)	19.0 (14.0–28.0)	18.0 (12.0–25.0)
WBC count (10^3^ cells/mm^3^), median (IQR)	6.7 (5.6–8.1)]	6.8 (5.5–8.1)	6.8 (5.7–8.3)	7.1 (6.0–8.5)	7.0 (5.7–8.5)

BMI: body mass index; ADPKD: autosomal dominant polycystic kidney disease; HIV: human immunodeficiency virus; AST: aspartate aminotransferase; ALT: Alanine aminotransferase; WBC: white blood cell.

The prevalence of analgesic prescriptions varied across different countries by CKD stage (Table 2). Analgesics were more often prescribed in the USA (38% overall, 41% stage 3, 36% stage 4, 39% stage 5) across all CKD stages, followed by France (24% overall, 25% stage 3, 24% stage 4, 21% stage 5) and Brazil (18% overall, 15% stage 3, 18% stage 4, 23% stage 5) (Table 2). NSAIDs were prescribed to a much greater proportion in the USA (24% stage 3, 17% stage 4, 15% stage 5) and in Brazil (14% stage 3, 18% stage 4, 23% stage 5) than in France (2% stage 3, 1% stage 4, 1% stage 5).

**Table 2:** Prevalence of analgesic prescriptions by country and CKD stage.

	Brazil			France			USA		
Characteristics	Stage 3	Stage 4	Stage 5	Stage 3	Stage 4	Stage 5	Stage 3	Stage 4	Stage 5
Patients, *n*	118	195	66	1389	1079	101	353	522	122
Any analgesic %	15	18	23	25	24	21	41	36	39
Opioids %	1	0	0	7	6	6	18	16	24
NSAID %	14	18	23	2	1	1	24	17	15
Other^a^ %	2	1	5	20	19	17	8	10	11

a Paracetamol, metamizole.

**Figure 3:**
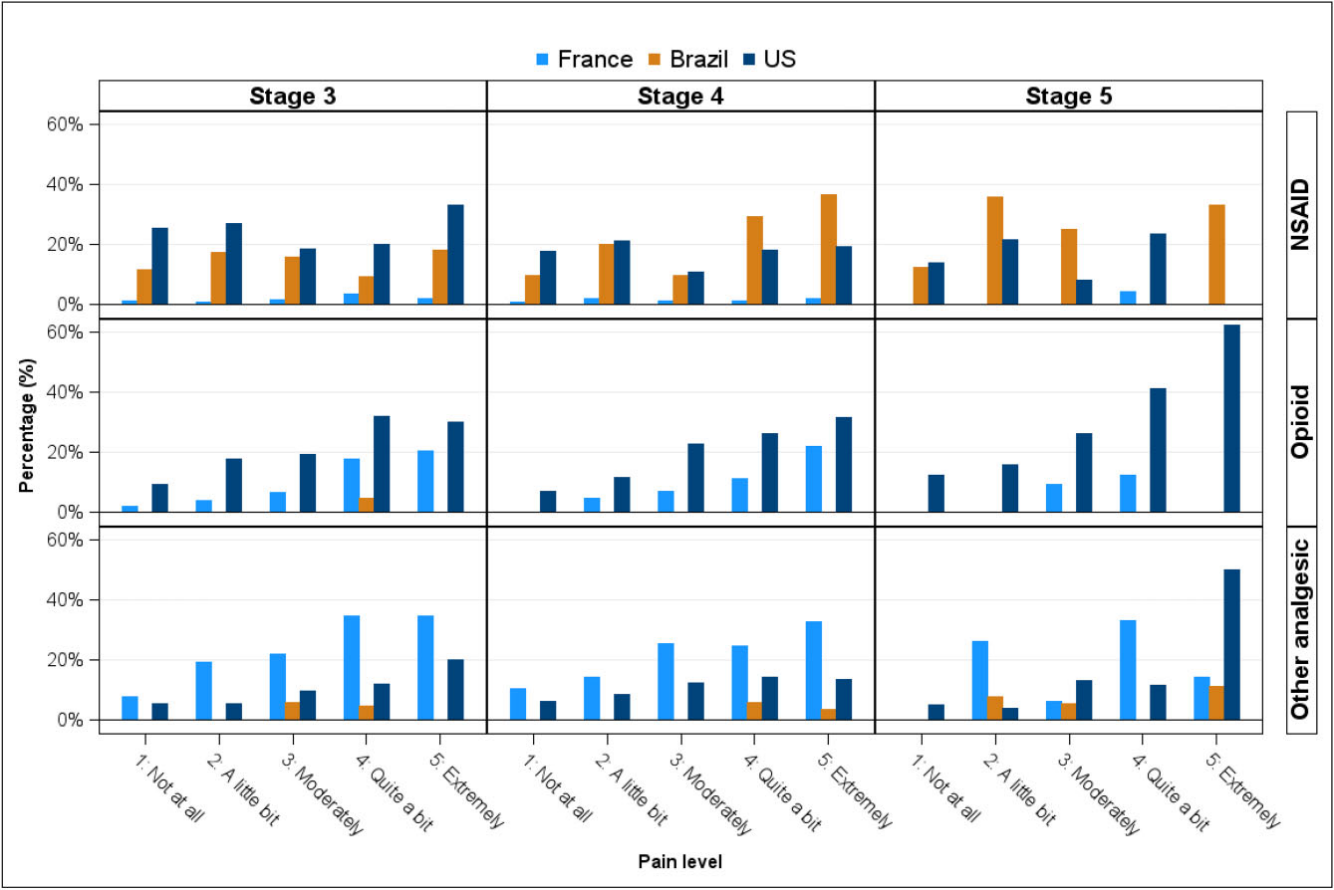
Prescription of NSAIDs or opioids by CKD stage and pain levels across countries.

Results stratified by CKD stage and country show clear differences across countries (Fig. 3, Supplementary Tables 1 and 2). The prescription of opioids was more common in the USA across CKD stages and pain levels compared with Brazil and France (Fig. 3). Patients with CKD stage 5 had a higher prevalence of opioid use across countries, with a much higher proportion of use in the USA (63%) compared with Brazil (no use) and France (22%). Even among patients who reported no relevant pain levels, the use of opioids was reported as 13% in the USA among stage 5 CKD patients, while in Brazil and France the prevalence was zero in this subgroup.

The frequency of NSAID prescriptions increased with greater severity of reported pain in all countries (Fig. 3). The frequency of NSAID prescriptions was remarkably lower in France for all levels of pain (0–4%) compared with Brazil (0–37%) and the USA (0–33%). In Brazil, 23% of stage 5 CKD patients had a prescription for NSAIDs, contrasting with nearly no prescriptions of such drugs in this stage in France or the USA.

Supplementary Table 3 describes patient characteristics by reported pain and analgesic use. Within the same level of reported pain, patients who were prescribed any analgesic tended to be female and to have more frequent psychiatric disorders. Among patients with non-skin cancer with extreme levels of pain, the use of any analgesic was higher than no use (20% versus 9%).

Worse levels of pain were correlated with poorer HRQOL. Patients more bothered by pain reported lower levels of general health, physical function and physical and emotional roles. Patients who were prescribed analgesics tended to report slightly lower QOL scores [15] (Table 3).

**Table 3:** 12-item Short Form Health Survey subscales by levels of pain and analgesic use.

Subscale	1 not at all		2 a little bit		3 moderately		4 quite a bit		5 extremely	
Analgesic use	No	Yes	No	Yes	No	Yes	No	Yes	No	Yes
Patients, *n*	920	177	729	247	730	301	378	246	119	98
General health	58.2 (19.5)	59.3 (20.6)	50.0 (19.3)	52.4 (20.9)	43.3 (21.0)	42.2 (21.1)	32.0 (21.5)	29.9 (18.8)	21.0 (23.1)	17.6 (20.8)
Physical function	82.3 (26.6)	80.1 (27.9)	66.2 (28.5)	62.1 (30.2)	51.9 (27.7)	46.9 (28.3)	30.6 (30.5)	28.9 (29.9)	21.5 (31.5)	23.4 (33.4)
Role physical	75.6 (29.9)	61.6 (37.7)	56.7 (27.4)	51.2 (30.9)	44.4 (23.3)	40.4 (23.0)	29.7 (25.2)	25.6 (22.9)	17.5 (25.1)	13.8 (23.3)
Role emotional	78.7 (27.4)	67.4 (34.9)	65.4 (27.8)	61.3 (32.2)	53.4 (26.4)	51.4 (27.0)	42.5 (31.2)	36.8 (29.2)	31.4 (34.1)	22.4 (27.9)

## DISCUSSION

In a patient population with moderate to advanced CKD under nephrology care, we found substantial variations in self-reported pain and its management across countries. Pain was more aggressively managed in the USA across all CKD stages and the use of NSAIDs was much lower in France compared with Brazil and the USA. The use of opioids was remarkably higher in the USA compared with other countries across all levels of reported pain and CKD stages. Finally, we found a clear correlation between the severity of pain and lower overall HRQOL, affecting both physical and mental domains.

Patients reporting more severe pain differed from those with lower levels of pain in our study. We found that female patients and those with diabetes, peripheral artery disease and neuropsychiatric disorders tended to report more intense levels of pain. These results are consistent with prior investigations limited to single countries and thus generalize the earlier findings to an international setting of moderate-to-advanced CKD patients [16]. Clinically, patients with diabetes and advanced CKD often have neurovascular complications that cause chronic pain [17]. Similarly, chronic and intense pain is often found in ND-CKD patients with symptomatic peripheral artery disease. Neuropsychiatric conditions in ND-CKD, pervasive as they are [18], frequently impact key patient-reported outcomes, including pain [19]. Finally, we found country differences in the frequency of self-reported pain. Patients in Brazil tended to report severe levels of pain more often than those in the USA and France.

Consistent with prior studies [15], we found a high prevalence of pain in this moderate-to-advanced CKD population. Despite being a common symptom, the lack of clear evidence to assist physicians in managing pain in ND-CKD is a challenge for improving patient-centred outcomes. From a global perspective, different policies and reimbursement processes across countries can also contribute to the challenge of alleviating pain in ND-CKD. Our results confirm our hypothesis that the prescription of analgesics could vary across countries, even within the same levels of reported pain. Strikingly, the use of opioids was much more liberal in the USA compared with France and Brazil, even among patients reporting mild levels of pain. These results are similar to those from studies reporting on global trends in opioid consumption [20] and practice patterns in diverse healthcare contexts, such as in surgery and primary care [21, 22].

A variation in the prescription of NSAIDs was also clear in our study. Notably, in Brazil, where opioids are rarely prescribed, the use of NSAIDs was up to 37% across CKD stages among patients with severe pain. These results contrast with the low utilization of NSAIDs in France and the USA in more advanced CKD stages. The prevalence of NSAID prescription in Brazil is higher than reported in a meta-analysis of observational studies carried out predominantly in high-income countries in Europe, Asia and the USA [16]. These results reinforce the striking variations in practice patterns in different regions of the world and may suggest disparities in access to safer interventions to manage severe pain in non-high-income countries [23]. The use of NSAIDs is contraindicated in advanced CKD stages because these drugs can cause acute kidney injury and hyperkalaemia and precipitate kidney failure and dialysis [24]. Additionally, NSAIDs increase the risk of cardiovascular and bleeding events, with higher risks among older patients with more comorbidities, common findings in the CKD population [24].

Notwithstanding the type of analgesic used, increased pain levels consistently correlated with poorer functional and HRQOL outcomes. To our knowledge, this is the first study to present the correlation between pain and HRQOL among ND-CKD patients. Prior studies have largely focused on populations with kidney failure undergoing haemodialysis. The urgent need for effective, multimodal pain management strategies in CKD patients is clear. While non-pharmacological interventions have been explored, their efficacy remains underinvestigated. Non-opioid medications for neuropathic pain, such as amitriptyline, desvenlafaxine or gabapentin, show promise, alongside novel drugs like VX-548, which inhibits nociceptor signalling with fewer side effects, though it has not yet been studied in CKD patients [25].

This study’s strength lies in its multinational data analysis, providing a comparative view of pain management practices across different healthcare systems using a standardized data collection process. We provide the first international comparison of practice patterns for pharmacological pain management while stratifying the results by severity of pain, a key patient-centred outcome. However, our study is limited by several factors. First, it is a cross-sectional descriptive study thus it is not designed to make causal claims nor to provide inferences regarding predictors of drug use or risk factors for adverse effects. Second, we did not have information on prescription dosages of analgesics thus we are unable to assess titration of drugs over time. Third, we did not include non-pharmacologic interventions, which are likely often prescribed for patients with CKD. Finally, since practice patterns of analgesic use vary around the world, our results can be affected by local, country or practice-level policies. We did not collect data regarding local practice policies for the prescription of analgesics in patients with CKD during the study.

In this international study describing practice patterns for analgesic prescriptions and self-reported pain and QOL among ND-CKD patients, we found clear differences in the prevalence of pain and its management across countries. The use of opioids is remarkably higher in the USA compared with France and NSAIDs are commonly prescribed in Brazil and the USA, even among advanced ND-CKD patients. We showed that pain is strongly correlated with poorer HRQOL and a high proportion of patients report extremely high levels of pain. Our findings highlight notable practice pattern variations that can be associated with adverse health outcomes for ND-CKD patients. Randomized controlled trials evaluating the effectiveness and safety of new strategies to treat pain are needed and international collaborations in the development of guidelines for pain management are warranted to mitigate practice patterns that may lead to adverse health outcomes in ND-CKD.

## Supplementary Material

sfaf346_Supplemental_File

## Data Availability

The data that support the findings of this study are available from Arbor Research Collaborative for Health, but restrictions apply to the availability of the data that were used for the current study and thus are not publicly available. However, data are available from the authors upon reasonable request and with permission of Arbor Research Collaborative for Health.
