# Supplementary material for: International variations in chronic kidney disease patients’ pain experience and its management
Source: Clin Kidney J. 2025 Dec 11;19(1):sfaf346. doi: 10.1093/ckj/sfaf346 (PMC12766450; doi:10.1093/ckj/sfaf346)
Supplement: sfaf346_Supplemental_File [file sfaf346_supplemental_file.docx]

**Supplementary Material**

**Supplementary Table 1.** Prevalence of **opioids** prescription by country, CKD stage, and of pain

|  | **Brazil** | | | **France** | | | **US** | | |
| --- | --- | --- | --- | --- | --- | --- | --- | --- | --- |
|  | **Stage 3** | **Stage 4** | **Stage 5** | **Stage 3** | **Stage 4** | **Stage 5** | **Stage 3** | **Stage 4** | **Stage 5** |
| **N** | 118 | 195 | 66 | 1389 | 1079 | 101 | 353 | 522 | 122 |
| **Pain interference with normal work** |  |  |  |  |  |  |  |  |  |
| **1: Not at all** | 0% | 0% | 0% | 2% | 0% | 0% | 9% | 7% | 13% |
| **2: A little bit** | 0% | 0% | 0% | 4% | 5% | 0% | 18% | 12% | 16% |
| **3: Moderately** | 0% | 0% | 0% | 7% | 7% | 9% | 19% | 23% | 26% |
| **4: Quite a bit** | 5% | 0% | 0% | 18% | 11% | 13% | 32% | 26% | 41% |
| **5: Extremely** | 0% | 0% | 0% | 20% | 22% | 0% | 30% | 32% | 63% |

**Supplementary Table 2.** Prevalence of **NSAID** prescription by country, CKD stage, and level of pain interference

|  | **Brazil** | | | **France** | | | **US** | | |
| --- | --- | --- | --- | --- | --- | --- | --- | --- | --- |
|  | **Stage 3** | **Stage 4** | **Stage 5** | **Stage 3** | **Stage 4** | **Stage 5** | **Stage 3** | **Stage 4** | **Stage 5** |
| **N** | 118 | 195 | 66 | 1389 | 1079 | 101 | 353 | 522 | 122 |
| **Pain interference with normal work** |  |  |  |  |  |  |  |  |  |
| **1: Not at all** | 12% | 10% | 13% | 1% | 1% | 0% | 25% | 18% | 14% |
| **2: A little bit** | 17% | 20% | 36% | 1% | 2% | 0% | 27% | 21% | 21% |
| **3: Moderately** | 16% | 10% | 25% | 2% | 1% | 0% | 19% | 11% | 8% |
| **4: Quite a bit** | 9% | 29% | 0% | 3% | 1% | 4% | 20% | 18% | 24% |
| **5: Extremely** | 18% | 37% | 33% | 2% | 2% | 0% | 33% | 19% | 0% |

**Supplementary Table 3.** Prevalence of **Other Analgesic*** prescription by country, CKD stage, and level of pain interference

|  | **Brazil** | | | **France** | | | **US** | | |
| --- | --- | --- | --- | --- | --- | --- | --- | --- | --- |
|  | **Stage 3** | **Stage 4** | **Stage 5** | **Stage 3** | **Stage 4** | **Stage 5** | **Stage 3** | **Stage 4** | **Stage 5** |
| **N** | 118 | 195 | 66 | 1389 | 1079 | 101 | 353 | 522 | 122 |
| **Pain interference with normal work** |  |  |  |  |  |  |  |  |  |
| **1: Not at all** | 0% | 0% | 0% | 8% | 10% | 0% | 5% | 6% | 5% |
| **2: A little bit** | 0% | 0% | 8% | 19% | 14% | 26% | 5% | 8% | 4% |
| **3: Moderately** | 6% | 0% | 5% | 22% | 25% | 6% | 10% | 13% | 13% |
| **4: Quite a bit** | 5% | 6% | 0% | 35% | 25% | 33% | 12% | 14% | 12% |
| **5: Extremely** | 0% | 3% | 11% | 35% | 33% | 14% | 20% | 14% | 50% |

* paracetamol, metamizole

**Supplementary Table 4.** Patient characteristics at baseline by level of pain interference and any analgesic use

|  | **1: Not at all** | | **2: A little bit** | | **3: Moderately** | | **4: Quite a bit** | | **5: Extremely** | |
| --- | --- | --- | --- | --- | --- | --- | --- | --- | --- | --- |
| **Analgesic use** | **No** | **Yes** | **No** | **Yes** | **No** | **Yes** | **No** | **Yes** | **No** | **Yes** |
| Patients, N | 920 | 177 | 729 | 247 | 730 | 301 | 378 | 246 | 119 | 98 |
| Age, years | 63.9 (14.2) | 69.8 (11.9) | 66.4 (13.0) | 70.1 (10.7) | 68.7 (12.4) | 70.5 (10.7) | 68.4 (11.9) | 69.9 (10.7) | 70.1 (12.8) | 70.1 (11.3) |
| Female, % | 35% | 38% | 37% | 38% | 39% | 50% | 43% | 53% | 56% | 59% |
| Body mass index, kg/m^2^ | 26.5 [23.9,29.9] | 28.6 [25.1,32.8] | 27.7 [24.5,31.2] | 28.9 [25.2,32.3] | 28.2 [25.0,32.3] | 29.5 [25.7,34.0] | 30.7 [26.2,35.5] | 30.8 [26.6,35.7] | 29.6 [25.3,35.2] | 33.4 [28.4,38.7] |
| eGFR, ml/min/1.73m^2^ | 31.1 (12.3) | 29.5 (10.9) | 30.3 (11.5) | 30.9 (12.2) | 30.0 (11.6) | 30.5 (11.0) | 29.3 (11.1) | 30.8 (11.6) | 27.5 (10.8) | 27.6 (10.2) |
| CKD stage, % |  |  |  |  |  |  |  |  |  |  |
| Stage 3 | 50% | 47% | 47% | 49% | 48% | 47% | 42% | 50% | 36% | 39% |
| Stage 4 | 43% | 45% | 47% | 43% | 44% | 47% | 51% | 42% | 52% | 50% |
| Stage 5 | 7% | 7% | 6% | 9% | 8% | 6% | 7% | 8% | 12% | 11% |
| Reported cause of CKD, % |  |  |  |  |  |  |  |  |  |  |
| Diabetes | 21% | 25% | 20% | 26% | 27% | 23% | 32% | 28% | 49% | 37% |
| Hypertension | 23% | 28% | 24% | 29% | 26% | 29% | 27% | 33% | 21% | 26% |
| Glomerulonephritis/ Vasculitis | 20% | 16% | 17% | 11% | 15% | 14% | 14% | 11% | 10% | 11% |
| Tubulointerstitial Disease | 13% | 8% | 12% | 13% | 11% | 15% | 10% | 10% | 10% | 15% |
| Polycystic | 6% | 7% | 7% | 5% | 5% | 2% | 2% | 4% | 5% | 6% |
| Other | 12% | 9% | 13% | 9% | 11% | 11% | 8% | 8% | 5% | 1% |
| Unknown | 4% | 7% | 7% | 6% | 5% | 6% | 8% | 6% | 1% | 4% |
| **Comorbidities, %** |  |  |  |  |  |  |  |  |  |  |
| Coronary artery disease | 18% | 25% | 24% | 28% | 27% | 29% | 36% | 33% | 31% | 41% |
| Cerebrovascular disease | 8% | 11% | 8% | 10% | 13% | 16% | 13% | 17% | 15% | 17% |
| Other cardiovascular disease | 18% | 26% | 21% | 26% | 28% | 31% | 28% | 31% | 33% | 34% |
| Peripheral vascular disease | 13% | 14% | 15% | 20% | 22% | 27% | 24% | 24% | 28% | 30% |
| Hypertension | 86% | 86% | 87% | 90% | 88% | 93% | 89% | 91% | 89% | 97% |
| Diabetes | 63% | 60% | 69% | 66% | 78% | 73% | 82% | 75% | 73% | 68% |
| Cancer (non-skin) | 19% | 21% | 20% | 17% | 23% | 22% | 21% | 22% | 9% | 20% |
| Gastrointestinal bleeding | 2% | 3% | 3% | 2% | 4% | 4% | 3% | 5% | 3% | 2% |
| HIV/AIDS | 2% | 1% | 0% | 1% | 1% | 1% | 1% | 1% | 0% | 2% |
| Lung disease | 6% | 9% | 8% | 14% | 13% | 11% | 13% | 19% | 16% | 16% |
| Neurologic disease | 3% | 5% | 3% | 4% | 4% | 3% | 3% | 6% | 13% | 5% |
| Any psychiatric disorder | 6% | 7% | 10% | 12% | 11% | 14% | 13% | 19% | 20% | 24% |
| Recurrent cellulitis/gangrene | 3% | 4% | 2% | 6% | 7% | 2% | 7% | 5% | 7% | 4% |
| **Laboratory** |  |  |  |  |  |  |  |  |  |  |
| Hemoglobin, g/dL | 13.0 (1.8) | 12.3 (1.8) | 12.8 (1.8) | 12.5 (1.8) | 12.7 (1.8) | 12.6 (1.7) | 12.4 (1.7) | 12.3 (1.8) | 12.1 (1.8) | 12.0 (1.5) |
| C-reactive protein (CRP), mg/dL | 2.8 [1.0,5.0] | 4.0 [1.1,6.0] | 3.3 [1.9,7.4] | 3.0 [1.5,7.2] | 3.8 [1.4,8.0] | 4.0 [2.2,9.5] | 5.0 [2.2,14.3] | 4.5 [2.9,12.0] | 5.0 [2.4,13.5] | 6.0 [2.8,13.1] |
| Aspartate Amino transferase (AST), Unit/L | 20.0 [16.0,27.0] | 19.0 [15.0,23.0] | 21.0 [16.0,26.0] | 20.0 [17.0,26.0] | 20.0 [17.0,26.0] | 20.0 [17.0,25.0] | 21.0 [16.0,25.0] | 21.5 [17.0,26.0] | 20.0 [16.0,30.0] | 21.5 [14.5,28.5] |
| Alanine Amino transferase (ALT), Unit/L | 18.0 [13.0,26.0] | 19.0 [12.0,24.0] | 19.0 [14.0,25.0] | 21.0 [15.0,27.0] | 18.0 [14.0,26.5] | 19.0 [13.0,25.0] | 18.0 [13.0,28.0] | 20.0 [14.0,27.0] | 16.0 [11.0,23.0] | 20.0 [13.0,29.0] |
| White blood cells count, 10^3^ cells/ mm^3^ | 6.7 [5.6,8.1] | 6.7 [5.6,8.0] | 6.8 [5.5,8.1] | 6.6 [5.5,8.2] | 6.9 [5.7,8.3] | 6.7 [5.7,8.2] | 7.2 [6.2,8.6] | 6.8 [5.8,8.4] | 7.0 [5.7,8.6] | 6.9 [5.7,8.4] |
